# Hepatic inflammation mediated by hepatitis C virus core protein is ameliorated by blocking complement activation

**DOI:** 10.1186/1755-8794-2-51

**Published:** 2009-08-08

**Authors:** Ming-Ling Chang, Chau-Ting Yeh, Deng-Yn Lin, Yu-Pin Ho, Chen-Ming Hsu, D Montgomery Bissell

**Affiliations:** 1 Liver Research Center and Department of Hepatogastroenterology, Chang Gung Memorial Hospital; Chang Gung University, College of Medicine, Taoyuan, Taiwan, Republic of China; 2 Liver Center and Department of Medicine, University of California, San Francisco, San Francisco, CA, USA

## Abstract

**Background:**

The pathogenesis of inflammation and fibrosis in chronic hepatitis C virus (HCV) infection remains unclear. Transgenic mice with constitutive HCV core over-expression display steatosis only. While the reasons for this are unclear, it may be important that core protein production in these models begins during gestation, in contrast to human hepatitis C virus infection, which occurs post-natally and typically in adults. AIMS: To more realistically model the effect of core protein production in the adult liver, we developed a mouse with conditional expression of HCV core and examined the effect of core protein production in the adult liver.

**Methods:**

Liver biopsy samples from transgenic mice with tetracycline(tet)-regulated conditional core protein expression were evaluated immunohistologically. Microarray analysis of HCV core transgenic mice with steatohepatitis pointed to a role of the complement pathway. This was further explored by blocking complement activation by in vivo administration of CD55 (decay accelerating factor for complement), which inhibits activation of C3.

**Results:**

Transgenic mice exhibited low, intermediate, or high HCV core protein expression when fed a permissive diet of standard chow. Aside from hepatic steatosis, hepatic inflammation and fibrosis were seen in mice with intermediate levels of core protein. Microarray analyses of inflamed liver demonstrated activation of both the complement (C3 up-regulation) and coagulation pathways (fibrinogen B up-regulation). Administration of CD55 reduced hepatic inflammation.

**Conclusion:**

Transgenic mice that conditionally express intermediate HCV core protein develop inflammation, steatosis, and fibrosis. These effects mediated by HCV core are reduced by administration of CD55, a regulator of the complement pathway. The model may be valuable in investigating the pathogenesis of liver inflammation in chronic hepatitis C.

## Background

Acute hepatitis C becomes chronic in at least two-thirds of cases. The infection typically is low-grade but does result in fibrosis and over a period of several years progresses to cirrhosis and liver failure in a significant proportion of cases [1]. The way in which the virus inflicts its long-term damage is not well understood. It is assumed that the inflammatory infiltrate, albeit often unimpressive, plays a role [2-5]. Much attention has been given also to intracellular effects of specific viral proteins, notably the hepatitis C virus (HCV) core protein [6-8]. HCV core has been shown in both experimental and human HCV infection to bind to endoplasmic reticulum and the membrane of lipid vesicles [9-11]. Core binding to mitochondria also has been shown in experimental models, with induction of oxidant stress [10,12]. These events may underlie hepatocellular steatosis and associated alterations in lipid metabolism, diabetes mellitus and insulin resistance [3].

Cells in culture transfected with *HCV core *are providing mechanistic insight into effects of the protein. However, observations in culture require in vivo confirmation. Also, inflammation cannot be studied in culture. The published in vivo models include several independently derived transgenic mouse lines with hepatocellular over-expression of HCV core. In most cases, the viral gene is controlled by a promoter that becomes active during gestation. Although the promoter is constitutive, by the time the mice are mature, core protein expression is usually low. Some mice display hepatic steatosis [13,14], but inflammation and fibrosis are absent. Interestingly, hepatocellular carcinoma develops beyond the age of 16 months in some mice [13-15].

Taken at face value, data from the previously published transgenic models suggest that core protein has little to do with inflammation in the HCV-infected liver. On the other hand, the timing – and like the level – of core expression are different from what occurs in human liver infected by HCV. The published data are limited but do indicate that HCV core protein is detectable by immunohistology in some chronic carriers [16]. To have an experimental model that more closely mimics these features of human hepatitis C, we generated mice with conditional expression of HCV core [9,10,17], using the tetracycline (tet)-off system [18]. By keeping the mice on a tetracycline-containing diet (chow compounded with doxycycline, or DOX), we completely suppress core expression during gestation and through weaning. When the mice are switched to a permissive diet (standard chow), HCV core appears within days, reaching easily detectable levels. The mice demonstrate not only steatosis but also inflammation and fibrosis, indicating that core protein expressed de novo in adult hepatocytes and at the appropriate level can cause the classical morphological changes of chronic hepatitis C. We show, moreover, that the complement cascade is involved in the hepatic reaction.

## Methods

### Transgenic mice

Mice conditionally expressing HCV core gene were generated as described [9,10,17]. Briefly, the HCV core gene (genotype 1b) was cloned into PUGH10-3 downstream of the tetracycline response element (TRE) [19]. Fertilized ova from FVB/N mice were injected with the construct. Founder mice were crossed with a second transgenic FVB/N line that is homozygous for the tetracycline transactivator (tTA) under control of the liver-enriched activating protein (LAP) promoter [18]. Unless otherwise indicated, mating pairs were maintained on doxycycline (DOX)-containing chow (200 mg/kg; Bio-Serve, Frenchtown, NJ) for suppression of HCV-core during development and through weaning. At approximately one month of age, DOX was withdrawn. Core expression was evaluated by liver biopsies taken at two months after birth. Five rearing designs (five mice for each rearing design) were used for the control mice to specify the HCV core protein effect: 1) double transgenic mice (DTM) that express both the HCV core and tTA were always fed DOX-containing chow; 2) other DTM were maintained on normal chow (*i*.*e*., no DOX); 3) single transgenic mice (STM) that express tTA only were fed DOX-containing chow until 1 month of age; 4), STM were maintained on DOX-containing chow all the time; and 5), other STM were maintained on normal chow. All mice were kept in specific pathogen-free rooms with regular quarantine. Only those that were serum-negative for common pathogen and viral markers were used in the experiments. Those with infectious agents, such as mouse hepatitis virus, mouse parvovirus, minute virus of mice, Reovirus-3, pneumonia virus of mice, epizootic diarrhea of infant mice, Theiler's murine encephalomyelitis virus, lymphocytic choriomeningitis virus, ectromelia, Sendai virus, sialodacryoadenitis virus, mycoplasma pulmonis, pinworms and fur mites, were excluded. The protocol was approved by Animal Care and Use Committee at Chang Gung Memorial Medical Center.

### Evaluation of the in situ HCV core protein level

The HCV core protein level was examined in situ in a wedge biopsy of liver, kidney, thymus, spleen, omentum, skin, heart, muscle, intestine, and lung. All biopsy specimens were obtained with the 2-month-old mice under isoflurane anesthesia. A portion of each organ was used for protein extraction and Western blot analysis, while the remainder was processed for histological study. HeLa cells carrying the same LAP-tTA (Clontech, Mountain View, CA) were transiently transfected with the TRE-HCV core construct in the absence of DOX. HeLa cell or mouse liver protein extracts were analyzed by Western blotting. After electrophoresis, proteins were transferred to a polyvinylidene difluoride membrane (Bio-Rad, Hercules, CA) and incubated with a 1:250 dilution of monoclonal mouse anti-HCV core antibody (Anogen, Mississauga, Ontario, Canada). After washes, membranes were incubated with secondary antibody (Bio-Rad), and developed using an ECL kit (Amersham, Buckinghamshire, UK). HCV core protein band intensity was quantified using a Fluor-S multiimager and Quality One software (Bio-Rad). Glyceraldehyde-3-phosphate dehydrogenase (Abcam, Cambridge, UK) was used as an internal control.

For immunohistochemical analysis, tissue samples were harvested, fixed in 4% paraformaldehyde, treated with 0.1% Triton X-100, incubated with HCV core protein antibody, and subsequently incubated with secondary antibody (Caltag, Carlsbad, CA). The background staining was eliminated by a mouse-on-mouse kit (Vector, Burlingame, CA).

### Evaluation of fatty liver

The oil-red-O stain for hepatic fat visualization was done with a commercial kit (Biogenex, San Francisco, CA).

### Evaluation of hepatic injury

For immunohistology, liver tissue samples were harvested and immediately fixed as described above for 12–24 h. Antibodies for fibronectin (Labvision, Fremont, CA), collagen (Biogenesis, Raleigh, NC), and alpha-smooth muscle actin (α-SMA) (Dako, Glostrup, Denmark) were applied followed by the respective second antibodies. The nuclei were counter-stained with methylene blue (Dako).

ALT was measured in tail blood using a Vitros DT60 II chemistry system (Johnson&Johnson, Rochester, NY). Frozen liver samples were homogenized and used to detect MDA by the thiobarbituric acid method (ZeptoMetrix, Buffalo, NY).

### Microarray analyses

Paired liver samples were studied in three experiments. In the first experiment (I), three DTM with high core expression were compared to three STM on the same diet regimen (DOX-containing chow until 1 month of age and then the permissive diet). In the second experiment (II), three DTM with intermediate core expression were compared to three STM on the aforementioned diet. In the third experiment (III), three DTM with intermediate core expression were compared to three DTM with high core expression using the same diet regimen. For all the experiments, the RNA were extracted from the livers of five pairs of 2-month-old female DTM versus 2-month-old female STM; and five pairs of 2-month-old male DTM versus 2-month-old male STM.

Total RNA was extracted and quantified as described previously [17]. 0.5 μg of total RNA was amplified by a fluorescent linear amplification kit (Agilent Technology) and labeled with Cy3-CTP or Cy5-CTP (CyDye; PerkinElmer, Waltham, MA) during the in vitro transcription process. RNA from the experimental mice was labeled with Cy5, and RNA from control mice was labeled with Cy3. Microarray analysis was performed according to a previously described protocol [17]. The microarray data have been deposited in the following website:  (accession number: GSE16403).

### Q-RT-PCR

The 30 genes with a known function and significantly increased or decreased expression from both experiments II and III (i.e., more than 1.8-fold in comparison with the control groups) were analyzed by Q-RT-PCR using the same RNA used for the microarray analyses. To prepare a cDNA pool from each RNA sample, total RNA (5 μg) was reverse transcribed using Moloney Murine Leukemia Virus reverse transcriptase (Promega, Madison, WI). Specific oligonucleotide primer pairs (appendix 1) selected from the Roche Universal Probe Library (Roche Corp, Castle Hill, Australia) were used for Q-RT-PCR. The specificity of each primer pair was tested using common reference RNA (Stratagene, La Jolla, CA) as the DNA template to perform Q-RT-PCR, followed by a DNA 500 chip run on a Bioanalyzer 2100 (Agilent Technology) to check the size of the PCR product.

Q-RT-PCR was performed on a Roche LightCycler 1.5 using the LightCycler^® ^FastStart DNA MasterPLUS SYBR Green I kit (Roche) as described previously [17].

### Injection of CD55 in transgenic mice with hepatic inflammation

Since the half-life of recombinant CD55 *in vivo *is around 24 hours and the recommended dose for rodents is 10 mg/kg [20], 250 μg of CD55 (R&D Systems, Minneapolis, MN) in 2.5 mL of phosphate-buffered saline were injected daily into the tail veins of five 2-month-old DTM with intermediate core expression. In our pilot study, serum ALT significantly decreased in the DTM after three daily injections (unpublished data). ALT assays and liver biopsies were performed before and 3 days after injection. Controls consisted of five 2-month-old DTM with intermediate core expression injected with the phosphate-buffered saline vehicle.

### Statistical analyses

Repeated measure analysis of variance (ANOVA) with Bonferroni correction for multiple comparisons were used to examine the time trends and group differences. Normalized microarray data were further filtered for missing genes and for genes exhibiting low expression levels. Significantly regulated genes (P < 0.05) represented expression levels that differed by at least 1.8. Considering multiple comparisons, the adjusted P values for the Benjamini and Hochberg method (false discovery rates; FDR) were also calculated for the selected genes, and an adjusted P value of less than 0.05 was chosen for subsequent functional and pathway exploration. Analyses were accomplished using the SAS 8.0 statistical package (SAS Institute, Cary, NC), and P < 0.05 was considered statistically significance.

Sex bias analysis was performed using the SAM [21], and P < 0.05 represented statistical significance.

Generic slim in GeneSpring version 7.3.1 (Agilent Technologies) was used for the functional category classification (Student's *t*-test was used in gene ontology (GO)). Potential pathways involved in fibrosing steatohepatitis were investigated using a web-based service (ArrayXPath; ), where Fisher's exact test and the FDR following Storey's scheme were applied to evaluate the statistical significance.

## Results

### Conditionally transgenic mouse lines exhibiting different levels of HCV core protein

**Figure 1:**
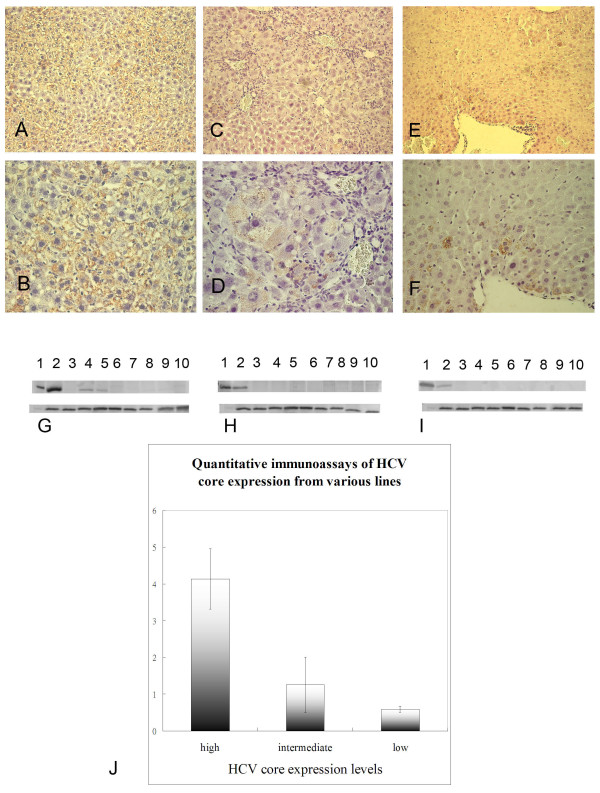
**Immunohistochemical stain for HCV core in the 2-month-old double transgenic mouse (DTM) liver with high (A, B), intermediate (C, D) and low (E, F) HCV core protein expression**. The upper region of panels G, H and I depict results of Western blots for HCV core protein extracted from the same livers after two months on control chow (permissive diet). Detection in DTM with high (G), intermediate (H), and low HCV core protein expression (I). Lane 1, HeLa cells transfected with HCV core plasmid (positive control, He); lane 2, liver (li); lane 3, heart (h); lane 4, kidney (k); lane 5, thymus (t); lane 6, omental fat (o); lane 7, lung (lu); lane 8, muscle (m); lane 9, intestine (i); lane 10, skin (s). As a loading control, the same samples were probed for GAPDH (lower panel G, H, and I). (J) Quantitative immunoassays for hepatic HCV core protein from high, intermediate, and low protein-expressing mouse lines. Y-axis, core bands densities (units) acquired from Fluor-S multiimager.

In the line exhibiting "high" HCV core protein, over 70% of the hepatocytes were immunohistologically positive (Figures 1A and 1B). A lobular gradient of positive cells was not observed. High-level core protein expression was confirmed by Western blot (Figure 1G, lane 2). In these mice, core protein was also detectable by Western blot, albeit at a very low-level, in the kidney and thymus (Figures 1G, lanes "K" and "t"). "Low" core protein expression was defined as < 10% positive hepatocytes (Figures 1E and 1F) and a proportionately diminished band on Western relative to "high" expressing liver, whereas "intermediate" core expression was defined as 20–30% positive hepatocytes (Figures 1C and 1D). Quantitative immunoassay of liver extracts revealed that liver with "high" core protein contained 4.135 ± 0.824 units, "low" contained 0.588 ± 0.079 units; "intermediate" expression (1.256 ± 0.756 units) fell between these two ("high" versus "moderate", *p *= 0.0034; " high" versus "low", *p *= 0.0001; "moderate" versus "low", *p *= 0.0078, each group n = 3) (Figure 1J).

### Degree of hepatic inflammation

**Figure 2:**
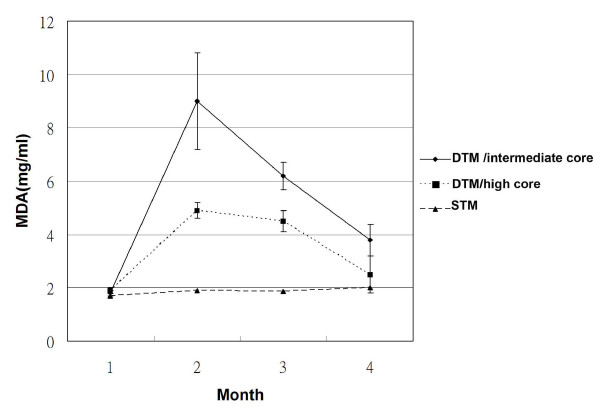
**Serum MDA levels for three male DTM with intermediate core expression, three male DTM with high core expression, and three STM in the first month (M1), second month (M2), third month (M3), and fourth month (M4) after one month of DOX withdrawal**. Data are expressed as mean ± SD.

The livers from all control mice were normal. The pathology for the DTM on permissive chow varied depending on the hepatic level of HCV core protein. Mice with high or low HCV core protein expression showed no significant inflammation (Figures 1A, B, E, and 1F). However, the liver from mice with intermediate HCV core protein expression exhibited an inflammatory infiltrate (Figures 1C and 1D) that was concentrated around hepatocytes displaying the core protein. The plasma MDA level was significantly higher in DTM with intermediate core expression than in DTM with high core expression, and exceeded the levels in STM (Figure 2) and either DTM or STM consuming DOX-containing chow (data not shown).

### Transgenic mice with hepatic inflammation exhibit hepatic steatosis and fibrosis

**Figure 3:**
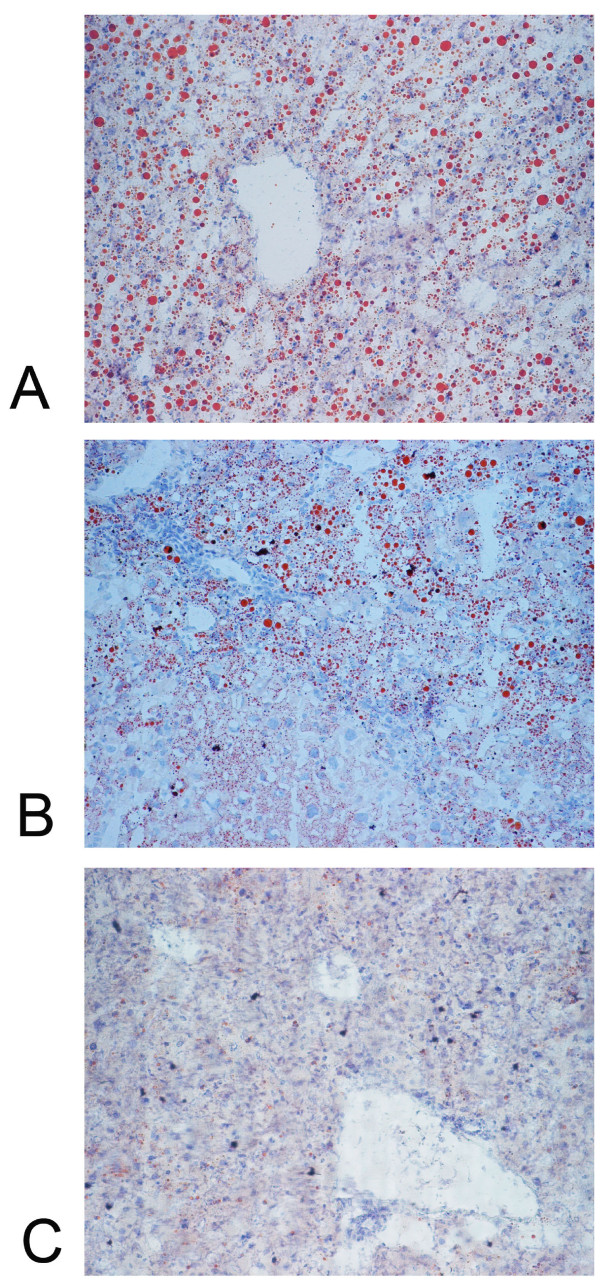
**Oil red stain for lipid in the 2-month-old DTM liver with high (A), intermediate (B) and low (C) HCV core protein expression**.

Oil red stains for the livers in DTM with high, intermediate and low HCV core expression are shown in Figure 3. The degree of steatosis paralleled the levels of HCV core expression (A, high; B, intermediate; C, low). The inflammatory cells were topologically associated with intrahepatic lipid in DTM with intermediate core expression (Figure 3B).

**Figure 4:**
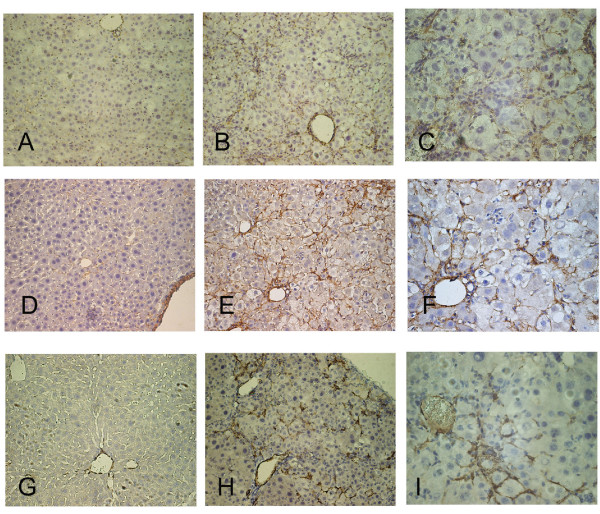
**Fibronectin staining in the liver of 2-month-old STM (A) and 2-month-old DTM with intermediate core expression (B, low-power field; C, high-power field)**. Collagen staining in STM (D) and DTM with intermediate core expression (E, low-power filed; F; high-power field). α-SMA staining in STM (G) and DTM with intermediate core expression (H, low-power filed; I; high-power field).

The DTM with intermediate HCV core expression also developed fibrosis by comparison with STM control mice (Fig. 4A, D and 4G). This was demonstrated by staining for fibronectin (Fig. 4B and 4C) and collagen (Fig. 4E and 4F). As evidence that the increase in matrix molecules represented de novo fibrogenesis, we also stained for alpha-smooth muscle actin (α-SMA), which is a marker of activated hepatic stellate cells [23]. Perisinusoidal cells staining for α-SMA were present and were topologically associated with inflammatory cells (Figures 4H and 4I).

### Microarray analyses

**Figure 5:**
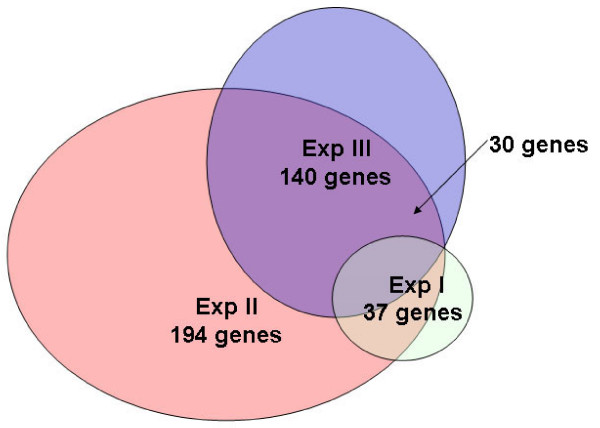
**Scheme for the gene numbers and associations of experiments I to III in the microarray analysis**. Exp. I, light green ellipse; Exp. II, pink ellipse; Exp. III, blue ellipse.

In experiment I (mice with HCV high core protein expression), 37 genes were considered differentially expressed in the liver in comparison to STM. Of these, 25 were upregulated and 12 were downregulated. In mice with intermediate core expression (experiment II), 194 genes were differentially expressed (120 were upregulated and 74 were downregulated) compared to the STM liver. Mice with intermediate core expression were compared to mice with high core expression (experiment III). In total, 140 genes were significantly altered: 98 genes were upregulated and 42 were downregulated. In a cross-comparison of data from experiments II and III, 30 genes were significantly changed (Figure 5, arrow). Significance Analysis of Microarrays (SAM) revealed no sex bias in gene expression from the microarray data.

The differentially expressed genes in each comparison were analyzed according to function (Table 1). The 30 dys-regulated genes from cross-comparison of data of experiments II and III were listed as Additional file 1.

**Table 1:** Gene functional analysis for selected genes from three experiments

**Category**	**Genes in category**	**Genes in category (%)**	**Selected genes in category**	**Selected genes in category (%)**	**P-value^†^**	**FDR_P^‡^**
**Experiment I**						
Biological Process						
GO:6953: acute-phase response	19	0.189	3	17.65	0.0000	< .0001
**Experiment II**						
Biological Process						
GO:9058: biosynthesis	853	8.492	26	20.47	0.0000	< .0001
Molecular Function						
GO:3824: catalytic activity	4096	34.96	73	54.07	0.0000	< .0001
GO:16740: transferase activity	1383	11.81	28	20.74	0.0020	0.022
GO:16491: oxidoreductase activity	570	4.866	14	10.37	0.0060	0.039
GO:30234: enzyme regulator activity	462	3.944	12	8.889	0.0071	0.039
GO:16829: lyase activity	113	0.965	5	3.704	0.0098	0.0431
**Experiment III**						
Biological Process						
GO:9058: biosynthesis	853	8.492	19	20.43	0.0003	0.0057
Molecular Function						
GO:16740: transferase activity	1383	11.81	25	26.04	0.0001	0.002
GO:30234: enzyme regulator activity	462	3.944	12	12.5	0.0004	0.0027
GO:3824: catalytic activity	4096	34.96	50	52.08	0.0004	0.0027

Genes in category: All genes belonging to a category

Genes in category (%): Genes belonging to the category as a percentage of the total number of genes analyzed (20,871)

Selected genes in category: Selected genes belonging to a category

Selected genes in category (%): Selected genes belonging to the category as a percentage of all genes belonging to that category

^†^Student's *t*-test.

^‡^Adjusted P value for Benjamini and Hochberg method (false discovery rate; FDR).

The major pathways altered within experiments I, II, and III are summarized in Table 2.

**Table 2:** Summary of the pathways identified from three experiments

**Pathway**	**P-value**	**q-value**
**Experiment I^a^**		
p53 signaling pathway, CCND1 and PCNA	0.0064	0.0362
small Leucine-rich Proteoglycan(SLRP) molecules, Lum	0.0468	0.1131
SREBP control of lipid synthesis	0.0236	0.0319
**Experiment II^b^**		
extrinsic prothrombin activation pathway, F5, F7, Fgb and Fga	0.001	0.004
intrinsic prothrombin activation pathway, F5, F9, Fgb and Fga	0.0015	0.0151
fibrinolysis pathway, serpine 1, Fgb and Fga	0.0016	0.0152
complement pathway	0.0017	0.018
inactivation of Gsk3 by AKT causes accumulation of b-catenin in alveolar macrophages	0.0024	0.0170
platelet amyloid precursor protein pathway, F9 and Serpine 1	0.0392	0.1788
α-synuclein and Parkin-medicated proteolysis in Parkinson disease, Snca	0.0455	0.1788
**Experiment III^c^**		
alternative complement pathway	0.0127	0.0644
lectin induced complement pathway	0.0158	0.0209
classical complement pathway	0.0174	0.0209
fibronolysis pathway	0.0174	0.0209
extrinsic prothrombin activation pathway	0.0205	0.0209
complement pathway	0.0252	0.0214

^a^ArrayXpath revealed 10 elements in 19 Biocarta pathways. Among the 19 pathways, three are significant.

^b^ArrayXpath has identified 29 elements in 44 Biocarta pathways. Among the 44 pathways, seven are significant.

^c^ArrayXpath revealed 2 (C3 and Fgb) elements in 38 Biocarta pathways. Among the 38 pathways, six are significant.

### Quantitative real-time polymerase chain reaction (Q-RT-PCR) analyses

As mentioned above, a total of 30 genes were simultaneously dysregulated in both experiment II and III and thus are implicated in the inflammatory response to HCV core protein. They were examined by Q-RT-PCR (Table 3). Seven displayed significant changes: Fabp (liver fatty acid binding protein) (M = log2 experimental/control = -1.07 ± 0.03), C3 (complement 3) (M = 3.6 ± 0.19), Saa1 (serum amyloid A1) (M = 1.57 ± 0.09), Fut 2 (fucosyltransferase 2) (M = 1.54 ± 0.05), Fgb (fibrinogen B) (M = -1.19 ± 0.05), lymphocyte antigen 6 complex, locus A (Ly6a) (M = 3.87 ± 0.16), and Ly6d (M = 4.6 ± 0.19). Five of the genes represented acute phase reactants: Fabl; C3; Saa1; Fut 2; and Fgb. The Ly6 family (also known as Sca-1) occurs on reactive cells, immune cells, as well as on hematopoietic stem cells.

**Table 3:** The M values of qPCR of the dys-regulated genes from cross-comparison of data from experiments II and III.

***Gene name***	***M values of qPCR***
Ly6d	4.6 ± 0.19
Ly6a	3.87 ± 0.16
C3	3.6 ± 0.19
Saa1	1.57 ± 0.09
Saa3	1.23+/-1.25
Fut2	1.54 ± 0.05
Fgb	-1.19 ± 0.05
Saa2	1.03+/-1.78
Calml4	0.67+/-0.54
Ly6i	0.89+/-0.45
Cte1(Acot1)	-2.12+/-2.8
Fabp1	-1.07 ± 0.03
Gstp1	0.62+/-0.83
Anxa5	0.55+/-0.45
Lcn2	0.67+/-0.66
Usp18	0.86+/-0.76
Lgals1	0.58+/-0.43
Igfbp1	0.46+/-0.88
Col3a1	0.43+/-0.77
Slc39a4	0.44+/-0.36
Gstm2	0.66+/-0.86
Ltb4dh	0.64+/-0.48
Anxa2	-0.58+/-0.7
Cyp4a14	-0.34+/-0.76
Sdf2l1	-0.67+/-0.55
G0s2	-0.45+/-0.56
Serpina4-ps1	-0.8+/-0.95
Hspa1a	-0.51+/-0.59
Hspca	-0.56+/-0.44

### Effect of CD55 injection on hepatic inflammation

**Figure 6:**
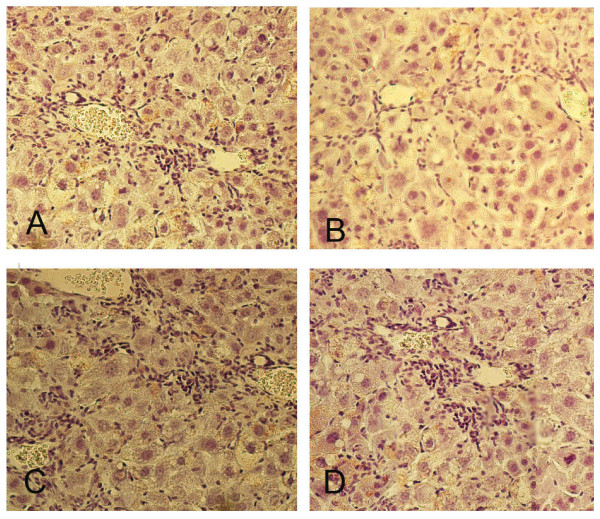
**Liver histology for 2-old-month DTM with intermediate core expression before (A and C) and after CD55 (B) or PBS (D) injection**.

Of the various proteins identified, the one most closely linked to human hepatitis C is C3 [24]. To probe the functional significance of this association, we studied the effect of a complement pathway inhibitor on HCV core protein-mediated hepatic inflammation. Mice were injected with CD55, which accelerates the decay of C3 convertases in the classical and alternate complement pathways [25]. In DTM with intermediate core expression, the injection of CD55 dramatically reduced hepatic inflammation (Table 4). The mean ALT levels for the transgenic mice 3 days after the CD55 injection were significantly lower than those of the mice that were injected with vehicle alone (179.2 ± 50.92 vs. 654.4 ± 134.51; *P *< 0.01). The histology of the livers from mice given a CD55 injection also showed fewer infiltrating inflammatory cells compared to mice that received PBS only (Figure 6).

**Table 4:** Effect of CD55 injection on hepatic inflammation

**HCV core transgenic mice exhibiting hepatic inflammation (s/p CD55 injection)**		**HCV core transgenic mice exhibiting hepatic inflammation (s/p PBS injection)**		
Day 1	Day 3	Day 1	Day 3	*P *value
723.4 ± 51.55^a^	179.2 ± 50.92^b^	731.4 ± 87.0^c^	651.4 ± 134.51^d^	0.0001

^a^Aminotransaminase (mean ± SD) of DTM with intermediate core expression prior to CD55 injection (day 1).

^b^Aminotransaminase of DTM with intermediate core expression after CD55 injection (day 3).

^c^Aminotransaminase of DTM with intermediate core expression prior to PBS injection (day 1).

^d^Aminotransaminase of DTM with intermediate core expression after PBS injection (day 3).

*P *value: for the difference of ALT (mean ± SD) between day 1 and day 3.

## Discussion

We report here several lines of mice with conditional expression of HCV core protein, which on the permissive diet differed with regard to the hepatic level of HCV core protein production. In tracking histological changes by serial biopsy, we found that some mice developed steatohepatitis, while others exhibited only bland steatosis. Interestingly, mice with the more advanced changes all had intermediate levels of HCV core protein in the liver. Mice with high or low levels of the protein had minimal findings – at most, steatosis without inflammation. The reasons for the lack of findings in mice with high expression are speculative but could include induction of immunological tolerance or inhibition of antigen presenting cells [26]. We reported previously that mice with relatively high expression of HCV core display markers of oxidant stress [9,10,17]. The synergistic effect of hepatic inflammation and core-related oxidative stress may lead to high MDA levels in these transgenic mice with intermediate core protein expression.

Gene profiling of liver demonstrated that changes in gene expression in mice with a high level of HCV core are limited to acute-phase responses (experiment I), whereas in mice with intermediate HCV core, the changes are much broader (experiments II and III). This is expected, given our observation that intermediate core protein expression is associated not only with steatosis but also with inflammation and fibrosis (experiments II and III). In the latter studies, expression of seven genes including C3 and Fgb, the coagulation pathway, and complement pathway activation were altered. Complement proteins are involved in early innate immune responses against pathogens and play a role in clearing circulating viral antigens from the blood of infected hosts [24]. The possibility of viral infectious hepatitis in those mice was excluded, because all the common pathogen and viral markers were serum-negative upon liver biopsy and no pathologic findings were evident in any control mouse. We propose that core protein is responsible for activating the complement pathway either directly or by inducing inflammation. The linkage between inflammation and coagulation has been noted [27]. The former impacts the initiation phase of blood coagulation [28]. Also, it has been reported that thrombin is an activator of hepatic stellate cells, stimulating their proliferation and fibrogenesis [29]. Therefore, we propose that HCV core initially elicits hepatic inflammation, which then activates the coagulation pathway. Hepatic fibrosis ensues, at least partly from the activation of the coagulation pathway.

We had shown high core expression leads to bland steatosis and C3 downregulation (experiment I) [17], in contrast to the findings in experiments II and III, suggesting that C3 is a critical determinant of hepatic inflammation in the HCV core-expressing liver. To test the functional importance of this change, we administered CD55 (decay-accelerating factor), which reduces the activity of C3 [25]. The liver of the treated mice exhibited a striking decrease in inflammation. Previous studies have documented alterations of the complement pathway in human hepatitis C; indeed, plasma C3 has been proposed as a marker for activity in this disease [24]. Furthermore C5, a related protein that acts downstream of C3 in the complement cascade, has been identified as conferring susceptibility to fibrosis in mice with liver injury from carbon tetrachloride and in humans with chronic hepatitis C. Inhibition of the C5a receptor has antifibrotic effects in murine fibrosis [30].

## Conclusion

In conclusion, the present study has shown that core protein is capable in vivo of eliciting inflammation of sufficient intensity and duration to give rise to fibrosis. While not excluding a role for other viral proteins, our data clearly demonstrate that core is a significant participant in HCV-related inflammation. The data suggest that interventions targeting HCV core could result in diminished hepatic inflammation, if not by interfering with viral assembly, then by blocking the ability of the core protein to stimulate the complement pathway.

## Abbreviations

(HCV): hepatitis C virus; (C3): complement 3; (MS): metabolic syndrome; (MRI): magnetic resonance imaging; (DEXA): dual-energy X-ray absorptiometry; (TRE): tetracycline response element; (tTA): tetracycline transactivator; (LAP): liver activator protein; (STM): Singletransgenic mice; (DTM): double transgenic mice; (PFA): paraformaldehyde; (GAPDH): Anti-glyceraldehyde-3-phosphate dehydrogenase; (HOMA-IR): Homeostatic model assessment for insulin resistance; (SBP): Systemic blood pressure; (Q-RT-PCR): Quantitative real-time polymerase chain reaction;(LRP5): low density lipoprotein receptor-related protein 5; (SAM): Significance Analysis of Microarrays; (FDR): false discovery rate; (SPSS): Statistics Package for Social Science;

## Competing interests

The authors declare that they have no competing interests.

## Authors' contributions

1) MLC made substantial contributions to conception and design, acquisition of data, analysis and interpretation of data and drafted the manuscript; 2) DMB was involved in drafting the manuscript and revising it critically for important intellectual content; and 3) CTY, DYL, YPH and CMH participated in the design of the study and performed the statistical analysis. All authors read and approved the final manuscript.

## Pre-publication history

The pre-publication history for this paper can be accessed here:



## Supplementary Material

Additional file 1 **30 dys-regulated genes in a cross-comparison of data from experiments II and III**. The data provided represent the M values from the analysis of microarray of the 30 dys-regulated genes. Click here for file
