# Supplementary material for: Hepatic inflammation mediated by hepatitis C virus core protein is ameliorated by blocking complement activation
Source: BMC Med Genomics. 2009 Aug 8;2:51. doi: 10.1186/1755-8794-2-51 (PMC2734540; doi:10.1186/1755-8794-2-51)
Supplement: Additional file 1 — 30 dys-regulated genes in a cross-comparison of data from experiments II and III. The data provided represent the M values from the analysis of microarray of the 30 dys-regulated genes. [file 1755-8794-2-51-S1.doc]

**30 dys-regulated genes in a cross-comparison of data from experiments II and III.**

| ***Function*** | ***Gene name*** | ***Description*** | ***Systemic name*** | ***M values of microarray*** |
| --- | --- | --- | --- | --- |
| [complement activation](http://www.ncbi.nlm.nih.gov/entrez/utils/fref.fcgi?http://amigo.geneontology.org/cgi-bin/amigo/go.cgi?view=details&depth=1&query=6956) | C3 | complement component 3 | NM_009778 | 3.374+/-0.184 |
| [protein binding](http://www.ncbi.nlm.nih.gov/entrez/utils/fref.fcgi?http://amigo.geneontology.org/cgi-bin/amigo/go.cgi?view=details&depth=1&query=5515) | Ly6d | lymphocyte antigen 6 complex, locus D | NM_010742 | 3.17+/-0.281 |
| [anchored to membrane](http://www.ncbi.nlm.nih.gov/entrez/utils/fref.fcgi?http://amigo.geneontology.org/cgi-bin/amigo/go.cgi?view=details&depth=1&query=31225) | Ly6a | lymphocyte antigen 6 complex, locus A | NM_010738 | 2.72+/-0.456 |
|  | 1810023F06Rik |  | NM_145449 | 2.05+/-0.432 |
| [ISG15-specific protease activity](http://www.ncbi.nlm.nih.gov/entrez/utils/fref.fcgi?http://amigo.geneontology.org/cgi-bin/amigo/go.cgi?view=details&depth=1&query=19785) PubMed 16139798 | Usp18 | ubiquitin specific protease 18 | NM_011909 | 1.67+/-0.345 |
| [oxidoreductase activity](http://www.ncbi.nlm.nih.gov/entrez/utils/fref.fcgi?http://amigo.geneontology.org/cgi-bin/amigo/go.cgi?view=details&depth=1&query=16491) | Ltb4dh | leukotriene B4 12-hydroxydehydrogenase | NM_025968 | 1.55+/-0.887 |
| [transporter activity](http://www.ncbi.nlm.nih.gov/entrez/utils/fref.fcgi?http://amigo.geneontology.org/cgi-bin/amigo/go.cgi?view=details&depth=1&query=5215) | Lcn2 | lipocalin 2 | NM_008491 | 1.48+/-0.343 |
| [glutathione transferase activity](http://www.ncbi.nlm.nih.gov/entrez/utils/fref.fcgi?http://amigo.geneontology.org/cgi-bin/amigo/go.cgi?view=details&depth=1&query=4364) | Gstm2 | glutathione S-transferase, mu 2 (Gstm2) | NM_008183 | 1.42+/-0.579 |
| transferase activity, transferring glycosyl groups | Fut2 | fucosyltransferase 2 | NM_018876 | 1.39+/-0.867 |
| [calcium ion binding](http://www.ncbi.nlm.nih.gov/entrez/utils/fref.fcgi?http://amigo.geneontology.org/cgi-bin/amigo/go.cgi?view=details&depth=1&query=5509) | Calml4 | calmodulin-like 4 | NM_138304 | 1.37+/-0.322 |
| [glutathione transferase activity](http://www.ncbi.nlm.nih.gov/entrez/utils/fref.fcgi?http://amigo.geneontology.org/cgi-bin/amigo/go.cgi?view=details&depth=1&query=4364) | Gstp1 | glutathione S-transferase, pi 1 | NM_013541 | 1.21+/-0.024 |
| [blood coagulation](http://www.ncbi.nlm.nih.gov/entrez/utils/fref.fcgi?http://amigo.geneontology.org/cgi-bin/amigo/go.cgi?view=details&depth=1&query=7596) | Fgb | fibrinogen beta chain | NM_181849 | 1.203+/-0.418 |
| [lipid transporter activity](http://www.ncbi.nlm.nih.gov/entrez/utils/fref.fcgi?http://amigo.geneontology.org/cgi-bin/amigo/go.cgi?view=details&depth=1&query=5319) | Saa2 | serum amyloid A 2 | NM_011314 | 1.20+/-0.088 |
| [lipid transporter activity](http://www.ncbi.nlm.nih.gov/entrez/utils/fref.fcgi?http://amigo.geneontology.org/cgi-bin/amigo/go.cgi?view=details&depth=1&query=5319) | Saa3 | serum amyloid A 3 | NM_011315 | 1.16+/-0.435 |
| [cytoskeletal protein binding](http://www.ncbi.nlm.nih.gov/entrez/utils/fref.fcgi?http://amigo.geneontology.org/cgi-bin/amigo/go.cgi?view=details&depth=1&query=8092) | Anxa2 | protein-tyrosine kinase substrate p36 (calpactin I heavy chain), complete cds. | D10024 | 1.13+/-0.034 |
| [lipid transporter activity](http://www.ncbi.nlm.nih.gov/entrez/utils/fref.fcgi?http://amigo.geneontology.org/cgi-bin/amigo/go.cgi?view=details&depth=1&query=5319) | Saa1 | serum amyloid A 1 | NM_009117 | 1.11+/-0.234 |
| [negative regulation of coagulation](http://www.ncbi.nlm.nih.gov/entrez/utils/fref.fcgi?http://amigo.geneontology.org/cgi-bin/amigo/go.cgi?view=details&depth=1&query=50819) | Anxa5 | annexin A5 | NM_009673 | 1.11+/-0.078 |
| [collagen fibril organization](http://www.ncbi.nlm.nih.gov/entrez/utils/fref.fcgi?http://amigo.geneontology.org/cgi-bin/amigo/go.cgi?view=details&depth=1&query=30199) PubMed 9050868 | Col3a1 | procollagen, type III, alpha 1 | NM_009930 | 1.08+/-0.836 |
| [galactose binding](http://www.ncbi.nlm.nih.gov/entrez/utils/fref.fcgi?http://amigo.geneontology.org/cgi-bin/amigo/go.cgi?view=details&depth=1&query=5534) | Lgals1 | lectin, galactose binding, soluble 1 | NM_008495 | 0.98+/-0.566 |
| [anchored to membrane](http://www.ncbi.nlm.nih.gov/entrez/utils/fref.fcgi?http://amigo.geneontology.org/cgi-bin/amigo/go.cgi?view=details&depth=1&query=31225) | Ly6i | lymphocyte antigen 6 complex, locus I | NM_020498 | 0.96+/-0.576 |
| [cellular zinc ion homeostasis](http://www.ncbi.nlm.nih.gov/entrez/utils/fref.fcgi?http://amigo.geneontology.org/cgi-bin/amigo/go.cgi?view=details&depth=1&query=6882) PubMed 14612438 | Slc39a4 | solute carrier family 39 (zinc transporter), member 4 | NM_028064 | 0.91+/-0.357 |
| [nucleotide binding](http://www.ncbi.nlm.nih.gov/entrez/utils/fref.fcgi?http://amigo.geneontology.org/cgi-bin/amigo/go.cgi?view=details&depth=1&query=166) | Hspa1a | heat shock protein 1A | NM_010479 | -1.74+/-0.556 |
| [fatty acid binding](http://www.ncbi.nlm.nih.gov/entrez/utils/fref.fcgi?http://amigo.geneontology.org/cgi-bin/amigo/go.cgi?view=details&depth=1&query=5504)* | Fabp1 | fatty acid binding protein 1, liver | NM_017399 | -1.588+/-0.213 |
| [oxidation reduction](http://www.ncbi.nlm.nih.gov/entrez/utils/fref.fcgi?http://amigo.geneontology.org/cgi-bin/amigo/go.cgi?view=details&depth=1&query=55114) | Cyp4a14 | cytochrome P450, family 4, subfamily a, polypeptide 14 | NM_007822 | -1.56+/-0.776 |
|  | Serpina4-ps1 | serine (or cysteine) proteinase inhibitor, clade A, member 4, pseudogene 1 | BC024071 | -1.41+/-0.878 |
| [positive regulation of nitric oxide biosynthetic process](http://www.ncbi.nlm.nih.gov/entrez/utils/fref.fcgi?http://amigo.geneontology.org/cgi-bin/amigo/go.cgi?view=details&depth=1&query=45429) | Hspca | heat shock protein 1, alpha | NM_010480 | -1.22+/-0.245 |
| [endoplasmic reticulum](http://www.ncbi.nlm.nih.gov/entrez/utils/fref.fcgi?http://amigo.geneontology.org/cgi-bin/amigo/go.cgi?view=details&depth=1&query=5783) | Sdf2l1 | stromal cell-derived factor 2-like 1 | NM_022324 | -1.13+/-0.221 |
| [acyl-CoA thioesterase activity](http://www.ncbi.nlm.nih.gov/entrez/utils/fref.fcgi?http://amigo.geneontology.org/cgi-bin/amigo/go.cgi?view=details&depth=1&query=16291) PubMed 11694534 | Cte1(Acot1) | cytosolic acyl-CoA thioesterase 1 | NM_012006 | -1.03+/-0.098 |
| [insulin-like growth factor binding](http://www.ncbi.nlm.nih.gov/entrez/utils/fref.fcgi?http://amigo.geneontology.org/cgi-bin/amigo/go.cgi?view=details&depth=1&query=5520) | Igfbp1 | insulin-like growth factor binding protein 1 | NM_008341 | -0.99+/-0.234 |
| [cell cycle](http://www.ncbi.nlm.nih.gov/entrez/utils/fref.fcgi?http://amigo.geneontology.org/cgi-bin/amigo/go.cgi?view=details&depth=1&query=7049) | G0s2 | G0/G1 switch gene 2 | NM_008059 | -0.89+/-0.221 |
